# The Week's Work

**Published:** 1911-03-04

**Authors:** 


### The Week's Work.

### THE NEW KING'S COLLEGE HOSPITAL.

The Building Committee was well advised in showing the representatives of the London Press over the partly-finished blocks of the new King's College Hospital at Denmark Hill. Everything points to the fact that this institution will be one in which not only the neighbourhood but London and the country at large can take a pride. A great hospital in the making is a wonder with which newspaper men should be more intimately acquainted than they are at present; it is only by knowing what the work means, what difficulties have to be overcome, what stumbling blocks cleared out of the way, that they can assist in furthering its completion and helping it to attain the height of excellence and modernity which should be the aim of hospital workers. Elsewhere in this issue we briefly refer to the construction of the new hospital, and we expressly refrain from criticism or comment because we are fully aware that it is impossible to do justice to those who are responsible for the plans and arrangement of the work until we have had a more convenient opportunity for going over the buildings at leisure. With the lay press the case is, of course, different. Its representatives are not concerned with the technicalities of the plans, and the little details of construction which interest hospital workers but are caviare to the lay public. For them last Saturday's inspection revealed one thing especially—the fact that there is arising in south-east London an institution that can stand comparison with the best of its kind.

### THE FUTURE.

Nothing could be more impressive than the unadorned simplicity of the buildings as seen on Saturday. Nothing, too, could press home more clearly the moral that if this hospital is to be what its architect designed it to be—a first-class modern hospital—the public must respond generously to the appeal for funds to carry it to final completion. Every effort is being made by the Committee to raise a further sum of £150,000 in order to enable the institution to be opened as soon as possible. The hospital will provide accommodation for 600 beds, and we know of no better method of commemorating the Coronation Year that wealthy Londoners can attempt than to endow one or more of these beds, and by so doing commemorate at the same time their generosity and their practical interest in the work that King's has done in the past and that it will undoubtedly continue to do, in a more thorough and up-to-date fashion, in the future. The Press can do much to bring this lesson home to reading London. It has seen the bare, unfurnished blocks, it has watched as it were the structure rising, like that gigantic temple fashioned by Mammon, not indeed with the sound of dulcet symphonies but with the clang of hammers and the creaking of cranes; it can do much to enable the Committee to invite it a few months hence, to see that structure fully furnished and ready for the reception of the suffering poor of South London. Not by an isolated reference to last Saturday's inspection so much as by constant reiteration of the needs of the institution will the Committee be best served by those who are in a position to bring its wants before the attention of the public.

### A POPULAR EXPOSITION OF HOSPITAL WORK.

We note with much satisfaction an article which appears in the current (March) issue of "The Sunday at Home," a periodical published at 4 Bouverie Street, E.C., on "The Hospitals of London: A Survey of the Whole Position, by a Special Commissioner." The article is ably and informatively written and the commissioner has taken pains to secure the latest statistics and to make his paper valuable by means of explanatory figures and diagrams. He deals in a careful and impartial fashion with the work that the hospitals of the metropolis are doing, and shows how important that work is, and what prodigious difficulties have to be contended with. Articles of this nature can only be productive of good. They are instructive and when written with such care as this they are worthy of every praise. At the present moment there is an organised attempt on the part of certain lay papers to discredit the administration of some of our best and most carefully managed hospitals. Doubtless that attempt is the result of a mistaken impression that by means of pertinacious and aggressive attacks upon these institutions the way will be cleared for certain reforms which are, so far as the papers we have in mind are concerned, exceedingly nebulous, but there can be equally little doubt that it will react injuriously upon the hospitals in question. The public knows too little about the inside working of our great hospitals to sift the false from the true in these allegations, and hospital workers will therefore hail with gratitude any endeavour on the part of the lay press to present the facts to their readers in a true condition. The article to which we allude does this in an able manner, and we hope that it will be widely read. We would like to suggest that it should also be reprinted and distributed in pamphlet form, as in that way its usefulness is likely to be greatly enhanced. Members of the Committees of our London hospitals might very well interest themselves in the matter, and call attention to the value of the article.

### THE INSTITUTION QUARTERLY."

We welcome another addition to the number of institutional journals already in existence in the appearance of "The Institution Quarterly," which is published by the Board of Administration of the State of Illinois and is under the able editorship of Dr. Frederick Wines. The third number of the first volume contains a mass of interesting information in the shape of special articles dealing with subjects that are of particular interest to institutional workers—taking the word institutional in its widest sense. A notable feature of the paper is the space that it devotes to sociological subjects. It is becoming increasingly recognised that the institutional worker has to devote more attention than he has hitherto done to sociological aspects of institutional work. We have attempted to provide for this welcome recognition by the publication of a monthly supplement wholly devoted to the discussion of such questions as are at the moment claiming the attention of medical men, and of those who are concerned with the administration of charitable institutions. The limited space that we are able at present to allocate to this important section forbids the full treatment of these subjects that is necessary for their adequate consideration, but we imagine that the new section has already found favour with many of our readers. The American Journal has less reason to regret the exigencies of space. Six pages are occupied with a report of the recent American Prison Congress—a wholly admirable summary of extremely interesting speeches and discussions which is deserving of attention by all who are interested in the matter of penal reform. Four pages are devoted to the American Institute of Criminal Law, with a detailed account of the propositions of the International Congress on the subject. The other articles in this excellent number deal with the State Conference of Charities, a State sanatorium for consumptives, a biography of Florence Nightingale, a description of a new colour vision test for children, a paper on the "Non-Inheritability of Disease," and other matters of lesser importance. At the end is a detailed report on the various State hospitals and charities, with statistics.

### FOR HOSPITAL READERS.

There are, however, three other articles, not enumerated in the above catalogue, which deserve special mention. The first is a well-written paper on "Hospital Hints," in which the various views expressed at the American Congress are collated, and with which is incorporated the best of Dr. Thompson's recent contribution to "The Century" on hospital administration. This is a class of paper which is, unfortunately, not common in institutional journals. It is a clear précis of the best, a condensation which is very valuable for the busy man who has no time to read in *extenso* everything that has been written on one or two special points in which he is interested, and as such we commend it to the attention of hospital workers. The second important essay is on "The Duplication of Ancestors," and is wholly of eugenic interest. We have been much interested in perusing this article, which gives many historical examples of duplication, accompanied by explanatory diagrams which elucidate the text in a very striking manner. The third article deals with the institutions of North Carolina. In this State there are ten State institutions: three hospitals for the insane (one for negroes), and various asylums, homes, epileptic colonies, and reformatories. It is interesting to find that the State authorities are contemplating the establishment of consumptive "camps," and that the law requires the separation of consumptives in the gaols and almshouses in North Carolina. The question of the provision of special "camps" for consumptives is one that in the near future will have to be considered in this country. We do not, unfortunately, possess the climate which makes it possible for such "camps" to be kept going winter and summer, as it is proposed that they should do in North Carolina, but the economic advantages of providing cheap accommodation for this class of patients is a factor which we cannot afford to overlook. Consumption sanatoriums are expensive luxuries, and it is well worth considering if we may not obtain somewhat similar benefits by an expenditure of far less money on canvas tents or simple barrack shelters of the Moabite type, and by the creation of "camps" at special centres. We look forward to the next number of "The Institution Quarterly," and congratulate Dr. Wines and his coadjutors on the excellence of the present issue.

### THIS WEEK'S DRUG MARKET.

Business has been somewhat quieter, but on the whole prices are well maintained. As was anticipated, the price of codeine his been advanced by sixpence per ounce; the rising tendency in the values of opium and morphine continues. High rates are still ruling for new season's cod-liver oil, but there are few buyers of quantity. Quicksilver has again been raised in price and the quotation is twenty-five per cent. higher than at the beginning of February. The salts of mercury have been advanced in price accordingly. Indian hemp is dearer in consequence of a statement that the rate of the export duty from India is to be raised. Cape aloes is slightly dearer, as also are buchu leaves. Siam gum benzoin still fetches high prices. Gamboge is tending somewhat dearer.

